# Telemedicine in Coeliac Disease: In‐Person Appointments Are Favoured by Patients With a Lower Education Attainment and Lower Household Income

**DOI:** 10.1111/jhn.70014

**Published:** 2025-01-27

**Authors:** Yvonne Jeanes, Lidia Orlandi, Humayun Muhammad, Sue Reeves

**Affiliations:** ^1^ School of Life and Health Sciences University of Roehampton London UK

**Keywords:** coeliac, dietitian, gluten, telehealth, telemedicine

## Abstract

**Introduction:**

A gluten‐free (GF) diet, the only treatment for people living with coeliac disease (CD), is challenging, and international guidelines highlight the valuable role of healthcare professionals in enabling self‐management. The study aimed to explore the acceptability of telephone and online video consultations for adults with CD.

**Methods:**

A cross‐sectional study consisting of an online and paper survey was promoted to adults with CD.

**Results:**

Data from 496 adults with CD (87% female, 96% White) are presented, and 44% were adhering to the GF diet. Over half (58%) were very confident in understanding food labels from supermarkets, whereas only 38% were very confident when shopping online. The largest proportion of patients preferred in‐person healthcare appointments for CD (44%), with 20% reporting no preference and 21% preferring telephone appointments. Only 15% preferred online video consultations; of these, 97% were confident with online technology. A higher proportion of patients from a lower household income requested ‘in‐person’ appointments compared with those with a higher income (65% vs. 45% (*p* < 0.01)). Likewise, 58% of patients without a degree qualification requested ‘in‐person’ appointments compared with 45% of degree‐educated patients (*p* = 0.027).

**Conclusions:**

We highlight a significant proportion of adults with CD prefer an in‐person appointment. The paper survey enabled the views of a broader range of digitally confident patients to contribute to the study. With a global shift towards telemedicine and online resources, access and digital literacy is an important consideration for equitable healthcare to optimize patient self‐management of the GF diet.

## Summary

- Nearly half (44%) of adults with coeliac disease would like in‐person healthcare appointments.
- In‐person appointments were favoured by patients with lower education attainment and lower household income.
- Digital access and digital literacy are important considerations for equitable healthcare to optimize patient self‐management of the gluten‐free diet.

## Graphical abstract

A UK study highlights that 44% of adults with coeliac disease would like in‐person healthcare appointments. In‐person appointments were favoured by patients with lower education attainment and lower household income. Access and digital literacy are important considerations for equitable healthcare to optimize patient self‐management of the gluten‐free diet.

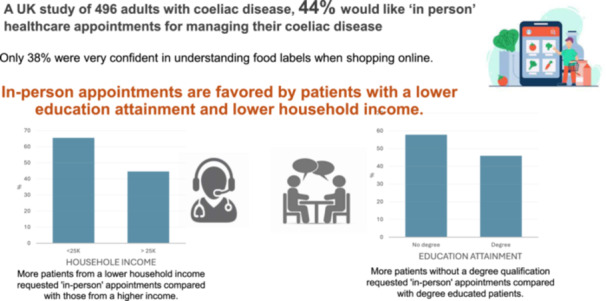

## 1 Introduction

Coeliac disease (CD) is an autoimmune disorder with a worldwide prevalence of approximately 1% [1]. Treatment requires strict adherence to a gluten‐free (GF) diet and, for the vast majority results in complete clinical and histological recovery. A GF diet excludes wheat, barley and rye; grains commonly used in breads, pasta, breakfast cereals, cakes, biscuits and pastries. Adherence to a GF diet is widely accepted as challenging; it requires the individual to gain knowledge and modify behaviours with external barriers, including a lack of widespread availability, high cost of GF foods and potential for cross‐contact eating out of the home environment [2, 3]. Adherence to a GF diet is highly variable and patients with CD report a treatment burden that is comparable to patients with end‐stage renal disease on dialysis [4]. Patients, especially in the first years after diagnosis, need expert dietetic follow‐up care to enable them to navigate their dietary options permitting them to adhere to a nutritionally adequate GF diet, minimize treatment burden and manage related social challenges.

There is consensus within international guidelines for follow‐up care to facilitate dietary adherence and minimize associated comorbidities [5, 6, 7]. Patients with CD have an increased risk of developing osteoporosis, vitamin deficiencies, other autoimmune conditions, small bowel lymphoma and adenocarcinoma [8, 9]. Suggested inclusions within healthcare follow‐up and reviews are anthropometrics, serology, assessment of dietary adherence, nutritional adequacy and treatment burden [5, 6, 7]. Studies have reported that discussion of symptoms and serology were the most frequently requested content for healthcare reviews by patients with CD [10, 11]. Multidisciplinary teams with expertise in CD have demonstrated improved coeliac‐related quality care metrics and symptoms [12]. Another study demonstrated how a specialist dietitian identified involuntary gluten consumption in 38% of adult patients with CD who self‐reported not consuming gluten, and thus was able to provide guidance on improving adherence and reducing persistent symptoms [13].

However, healthcare provision for adults with CD, at diagnosis, follow up and annual review varies greatly within and between countries; both in who the healthcare professional is and what is offered. In the United Kingdom, dietetic provision for CD depends on where the patient lives; it could be an in‐person one‐to‐one appointment(s), group education session(s), telephone appointment(s), questionnaires and/or online interfaces, such as video appointments or asynchronous online dietitian‐led content [11, 14]. It is important to recognize that, despite the guidelines, only a third of patients receive CD reviews in the United States and just a quarter in the United Kingdom [11, 15, 16]. From a UK cohort, 85% of adults with CD considered healthcare reviews important, these patients had significantly lower health literacy, greater dietary burden, poorer GF dietary adherence and lower GF food knowledge compared with those who did not consider reviews important [11].

Traditional healthcare provision, prior to the global COVID‐19 pandemic, predominately involved in‐person clinics within a hospital setting [17]. A qualitative study of adults with CD, participants reported experiencing difficulty in taking time off work and frustrations with hospital parking [18]. Due to the ‘stay at home’ guidance during the COVID‐19 pandemic, alternatives to in‐person appointments were rapidly developed, with telephone and online video consultations being delivered by healthcare professionals globally [19, 20]. Healthcare services being delivered online was not a new concept, it was the rapid and global shift that was unprecedented. Telemedicine has existed for many years and generally refers to the use of technology (telephone or online videoconferencing) to communicate with patients and provide healthcare at a distance [21]. An Italian study, mid‐pandemic, reported 86% were happy with telemedicine appointments, while 14% were unhappy and felt they were not looked after [19]; the timing of the survey responses may have been impacted by the perceived safety of in‐person appointments. Studies prepandemic also reported a patient preference for telephone appointments to save them time and money compared with an in‐person appointment [18, 22]; studies included reference to dietetic, consultant gasrtoenterologist, nurse and general practitioner appointments. Muhammad et al. reported an intervention demonstrating that a telephone appointment is effective in improving GF dietary adherence [23]. Telemedicine has the potential to be cost‐effective in improving patient care with its use continued in many aspects of healthcare since the COVID‐19 pandemic.

It is important to recognize the difference between in‐person and telephone/online interactions, not just the potential benefits of telemedicine, but also the challenges such as unreliable network connection, patients who lack technology access and/or technology skills and diminished client‐practitioner interaction and communication [24]. There is a real concern that telemedicine may inadvertently exacerbate health inequalities for susceptible populations, particularly those with limited digital literacy or restricted access to digital resources. Patients are diverse in their needs and preferences and thus service provision needs to reflect local population needs. Given there is a population of adults living with CD who do not receive healthcare reviews, it is important to hear from them when designing healthcare provision. The study aimed to explore the acceptability of telephone and online healthcare appointments in adults with CD.

## 2 Materials and Methods

A UK‐wide cross‐sectional online and paper‐based survey was designed to explore access to healthcare provision, experience of telehealth and views on future healthcare for CD. The survey design had input from gastroenterology specialist dietitians. Questions included diagnosis, demographics, understanding of GF diet and GF dietary adherence, and interaction with healthcare professionals for the management of CD, with areas for free comments to capture additional views of participants. GF dietary adherence was assessed by the validated Coeliac Disease Adherence Test (CDAT) score [25]; it consists of seven items on a Likert scale, and the sum of the numeric values provides a score ranging from 7 to 35; lower scores reflecting better adherence to a GF diet. Kurppa et al. reported a score of < 13 accurately predicts adequate adherence taking the assessment of an expert dietitian as the gold standard [26]. Experience of telemedicine questions was informed by those from a survey conducted to assess patient satisfaction with video teleconsultation in a virtual diabetes outreach clinic [27]. The full survey can be found in the Supporting Information; example questions include: ‘Have you attended a telephone or online video appointment for coeliac disease, or other health conditions, within the last 2 years?’ and ‘What type of appointment would you prefer to help you follow a gluten‐free diet/manage your coeliac disease in the future? e.g. in person, telephone, online video appointment’. Ethical approval was given through the procedures of the University of Roehampton Ethics Committee.

Inclusion criteria: participants needed to be aged ≥ 18 years, report a diagnosis of CD by a healthcare professional and have sufficient understanding of English to complete the survey.

During 2021, using online survey software (Jisc), the survey link was posted on social media and via email to Coeliac UK members and a customer database of a manufacturer of GF foods in the United Kingdom. We focused recruitment on people who may be less digitally confident via a paper survey aiming to reduce overall respondent bias. In 2022, Coeliac UK members without an email address on their membership application (*n* = 350) received a copy of the paper survey in the post with a prepaid envelope for return; 150 completed responses were received, a response rate of 43%.

Data was analysed using SPSS statistical package version 29 (IBM Corp.). Continuous data was tested for normality of distribution using the Kolmogorov−Smirnov test. Statistical significance was determined using the Independent *T* Test for continuous variables and the *χ* ^2^ test for categorical variables. *p* < 0.05 was adopted for indication of significance.

## 3 Results

### 3.1 Participant Descriptives

From 496 respondents (87% female, 96% White) there was representation from across the United Kingdom (Table 1). The average age of participants was 49 ± 21 years, with a greater percentage of > 70‐year‐olds completing the paper survey compared with the online survey (Table 1). From the online survey, 53.8% were current members of Coeliac UK, 26.6% had previously been a member and 19.7% were not members, compared with the paper survey (recruited via Coeliac UK) whereby 99% were current members and 1% had been a member.

**Table 1:** Study population descriptors of adults with coeliac disease.

	Online survey	Paper survey	All responses
Number of participants	346	150	496
Sex, *n* = (%)			
Male	20 (5.8)^a^	45 (30.0)^a^	65 (13.1)
Female	326 (94.2)	104 (69.3)	430 (86.7)
Unknown	0	1 (0.7)	1 (0.2)
Country of residence, *n* = (%)			
England	308 (89)	127 (84.7)	435 (87.7)
Scotland	22 (6.4)	8 (5.3)	30 (6.0)
Wales	10 (2.9)	7 (4.7)	17 (3.4)
Northern Ireland	6 (1.7)	6 (4.0)	12 (2.4)
Unknown	0	2 (1.3)	2 (0.4)
Ethnicity, *n* = (%)			
White	331 (95.7)	146 (97.3)	477 (96.2)
Asian/Asian British	7 (2.0)	1 (0.7)	8 (1.6)
Black^b^	1 (0.3)	0	1 (0.2)
Mixed/Multiple ethnic groups	6 (1.7)	0	6 (1.2)
Unknown	1 (0.3)	3 (2.0)	4 (0.8)
Age (years): Mean ± SD	38.9 ± 14.8	74.0 ± 10.0	49.4 ± 21.0
*n*=	344	146	490
*n* = (%) > 70 years	16 (4.6)^a^	107 (71.3)^a^	123 (24.8)
Highest level of education Total responses *n* = 463 *n* = (%) University degree	207 (62.0)^a^	19 (14.7)^a^	226 (48.8)
Annual income, *n* = (%)			
< £25 K	57 (16.5)^a^	76 (50.7)^a^	133 (26.8)
25–60 K	138 (39.9)	29 (19.3)	167 (33.7)
> 60 K	95 (27.5)	7 (4.7)	102 (20.6)
Unknown	56 (16.2)	38 (25.3)	94 (19)
Participant time commitment (*n* = 498)			
Full time employment	206 (59.5)	7 (4.7)	213 (42.9)
Part time employment/unpaid carer	64 (18.5)	11 (7.3)	75 (15.1)
Full time education	13 (3.8)	0	13 (2.6)
Retired	35 (10.1)^a^	116 (77.3)^a^	151 (30.4)
Full time unpaid carer/looking after the family/home	11 (3.2)	0	11 (2.2)
Long term disabled	11 (3.2)	12 (8.0)	23 (4.6)
Unemployed	5 (1.4)	2 (1.3)	7 (1.4)

^a^ Significantly different *p* < 0.001 by *χ* ^2^ analysis.

^b^ Black/African/Caribbean/Black British.

A higher proportion of the participants who completed the online survey were university educated, and in full time employment (Table 1). Participants who completed the online survey reported better access to good quality internet at home and a greater proportion were very confident in online technology, compared with those who completed the paper survey (Table 2). Thus, through the inclusion of the paper survey, the study has captured the views of a more representative cohort of adults living with CD, compared with online data collection alone.

**Table 2:** Participant access to technology and self‐management of coeliac disease.

	Online survey *n* = 346	Paper survey *n* = 150	All responses *n* = 496
*Access to technology*			
Internet access in the home, sufficient for video calls^b^	95^a^	47^a^	80
Phone‐only internet access	2	6	3
Physical, sensory or cognitive disabilities that make video/telephone communication difficult	3^a^	20^a^	8
Confidence in using online technology			
Very	71^a^	8^a^	52
Fairly	25	28	26
Not/Not at all confident	1.5^a^	46^a^	15
*Self‐management of coeliac disease*			
Knowledge of gluten‐free diet			
Good or excellent	93	91	92
Confidence in following a GF diet			
Very	59	63	60
Fairly	35	35	35
Not confident	1.7	0	1.2
Confidence in food labels – physical stores			
Very	61	51	58
Fairly	35	43	37
Not confident	2	1	1
Confidence in food labels –online stores^c^			
Very	43^a^	13^a^	34
Fairly	43	25	38
Not confident	4	10	6
Adherence to GF diet^d^			
CDAT < 13	40^a^	57^a^	4

^a^ Significantly different *p* < 0.01 by *χ* ^2^ analysis.

^b^ Includes desktop, laptop or tablet. Knowledge and confidence are self‐reported.

^c^ Paper survey *n* = 86; total *n* = 432.

d Paper survey *n* = 139; total *n* = 485.

Participants, irrespective of the format of the survey completed, had similarly high levels of self‐reported knowledge and confidence in managing CD with a GF diet (Table 2). The majority of all participants were confident in understanding food labels from physical shops/supermarkets, a lower proportion were confident in food labels when shopping online (Table 2).

### 3.2 Experience of Healthcare Appointments: In Person, Telephone and Online

The majority of participants (74%: 367/496) had participated in a telephone appointment for a health condition with 28% (*n* = 139/496) having one for CD within the last 2 years. Whereas only 9% (*n* = 42/496) had participated in an online healthcare appointment for any condition and 5% (*n* = 23/496) had one for CD. Ninety‐four participants (19%) reported not participating in a telephone nor online appointment for any healthcare reasons, 84% of those were not offered and 3% (*n* = 3) chose not to attend (missing data for 13%).

Forty‐one participants indicated they had a physical, sensory or cognitive disability that made video/telephone communication difficult, of these 75% reported having participated in a telephone appointment for a healthcare condition, with 34% (*n* = 14) having had one for CD.

**Figure 1:**
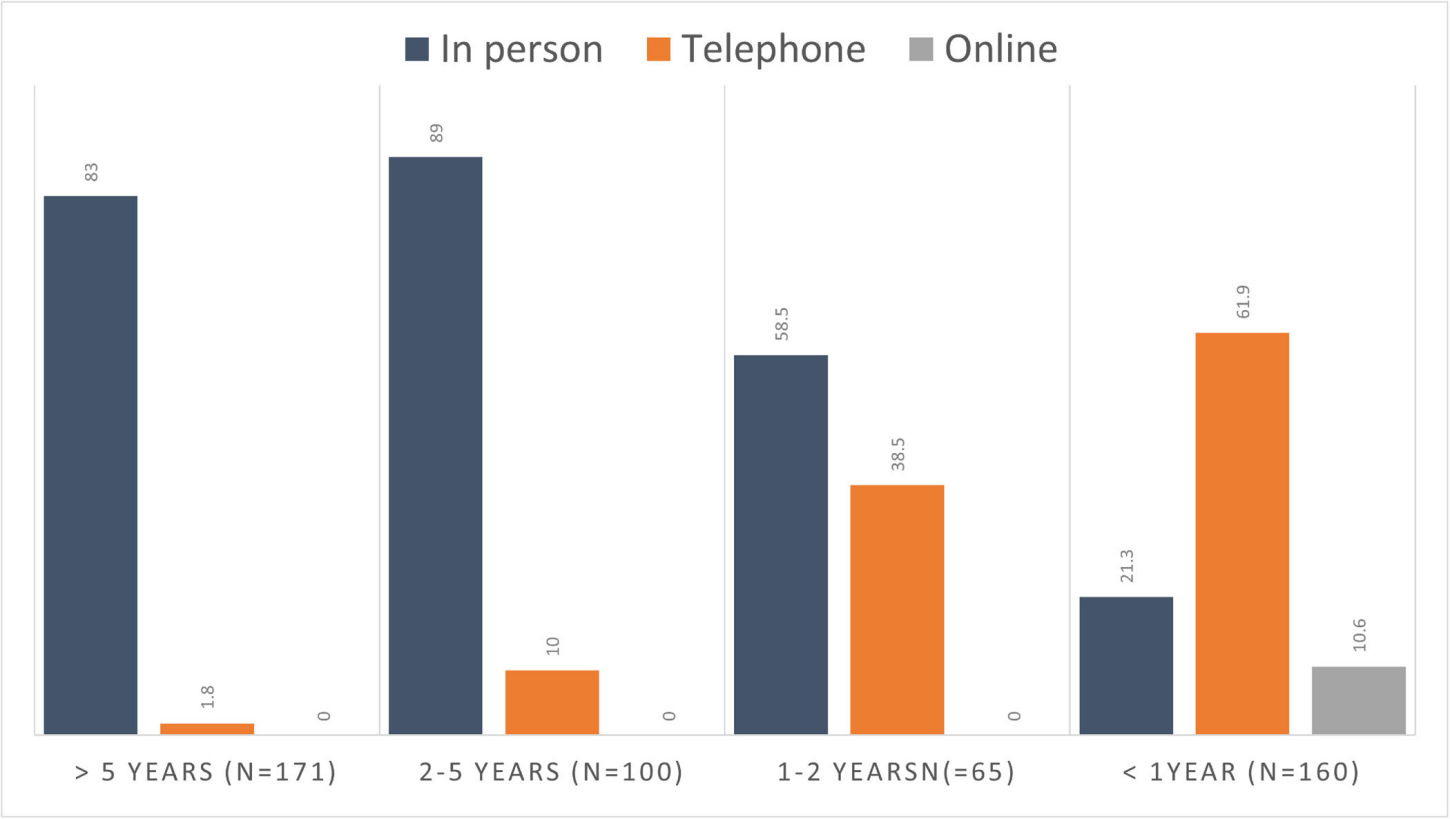
Percentage of in‐person, telephone or online appointments for coeliac disease (data collection 2021/22).

Figure 1 shows an increase in the proportion of telephone and online appointments for CD over time (the question was on their most recent appointment for CD: 32% was < 1 year ago, 13% 1–2 years, 20% 2–5 years and 35% > 5 years or never).

Three quarters (76%) who have experienced a telephone appointment for a health condition were satisfied with the sound quality, though 29% had distractions in their environment during their telephone appointment (Table 3). While only 35% agreed a telephone appointment saved them money, 62% agreed it saved them time (Table 3).

**Table 3:** Experience of telephone healthcare appointments by adults with CD.

	Online survey		Paper survey		Total	
Statements	Agree (%)	Disagree (%)	Agree (%)	Disagree (%)	Agree (%)	Disagree (%)
I was satisfied with the quality of the sound.	76	13	79	8	76	12
There were distractions during the appointment.	32	54	11	71	29	56
I was confident that the healthcare professional could assess my condition as if I was there.	36	43	47	32	38	41
Telephone call enables me to save money.	33	34	48	29	35	33
Telephone call enables me to save time.	61	29	63	24	62	22

*Note:* Agree/Strongly: Agree, Disagree/Strongly disagree responses combined. Remaining % responses were neither agree/disagree.

### 3.3 Preferences for In‐Person, Telephone or Online Appointments for CD

Annual healthcare review appointments were considered important for following a GF diet and managing the CD by 72% of survey participants, with a further 12% unsure having not been offered an annual review in recent years, only 16% indicated that they did not consider annual reviews important.

Forty‐four percent (204/463) of participants reported a preference for in‐person healthcare appointments for CD, 20% would be happy with either a telephone or an in‐person appointment and 21% had a preference for a telephone appointment. Sixty‐nine participants (15%) indicated a preference for online consultations; of these, 97% reported good internet access at home and 97% were confident with online technology. Time of diagnosis did not impact preference of appointment type: in‐person appointments were similarly preferred if patients were diagnosed in 2020 or 2021 compared with patients diagnosed before 2020 (55% vs. 49% respectively, *p* = 0.6). The preference for appointment type was not related to the proportion of patients adhering to a GF diet (% adhering according to the preference for appointments to be in‐person: 39%, either telephone or in‐person: 43%, telephone: 47%, online: 43%, *p* = 0.13).

**Figure 2:**
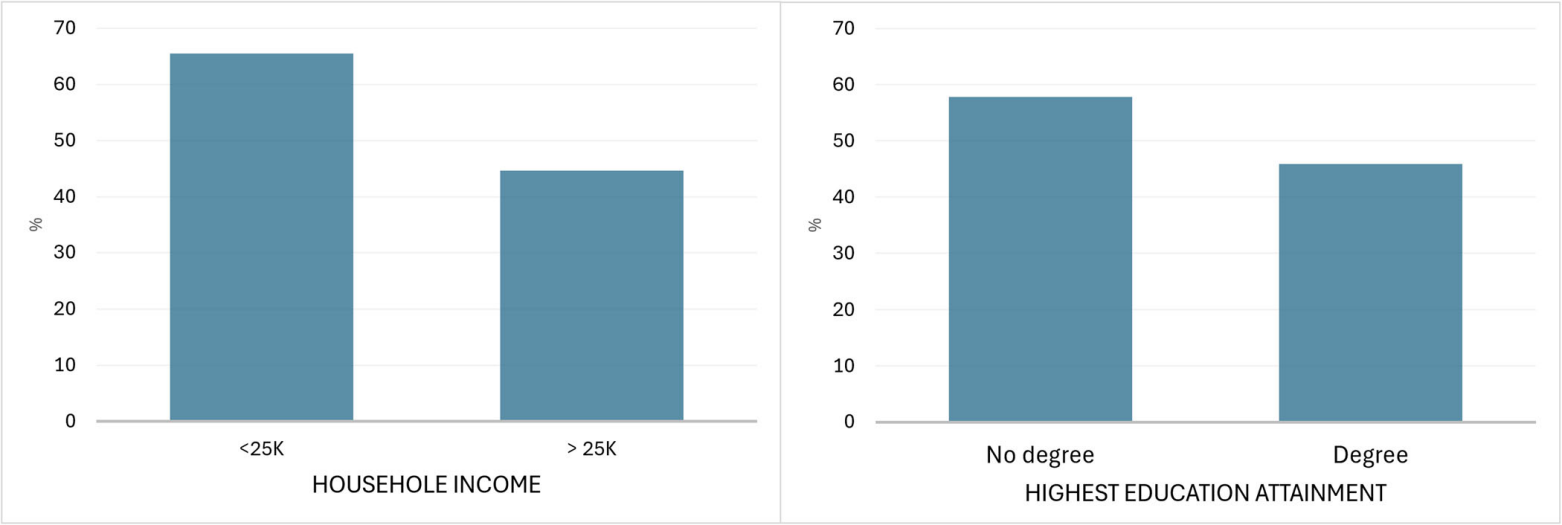
Significant preference for in‐person appointments split by household income and highest education attainment.

A significantly higher proportion of patients from a lower household income (< £25 K) preferred in‐person appointments (59/91) compared with those from a higher income (106/241) (65% vs. 44%; *p* < 0.01); illustrated in Figure 2. Additionally, a significantly greater proportion of patients without a degree (104/182) preferred an in‐person appointment compared with degree‐educated patients (93/205) (57% vs. 45%; *p* = 0.027). Age and confidence in technology did not make significance; 61% of patients aged > 70 years (42/69) requested an ‘in‐person’ appointment compared with 48% of participants < 70 years (159/331) (*p* = 0.052). Half of the participants (49%; 163/336) who reported confidence in technology requested an ‘in‐person’ appointment compared with 60% (39/66) of those reporting they were not confident with technology (*p* = 0.15).

## 4 Discussion

Our study indicates that self‐reported knowledge and confidence to manage the GF diet was high across the study group; however, confidence in understanding food labels for online shopping and using online technology for healthcare appointments was variable. With a global shift towards online resources, digital literacy and access become an important consideration for accessing equitable healthcare [25]. Healthcare appointments help enable patients to follow a GF diet, improve nutritional adequacy and importantly minimize treatment burden [6]. Regular and adequate follow‐up is needed to treat disease‐related symptoms, and identify and prevent possible complications related to CD [6]. Our study reports in‐person appointments were preferred by adults with CD who had a lower household income or adults without a degree qualification.

The expected increased use of telephone and online video appointments over time is presented in Figure 1, with 76% of participants having experience with telephone healthcare appointments. Prepandemic data from the United Kingdom reported that 70% of dietetic provision for CD was by in‐person appointments and 16% of dietetic departments were offering telephone appointments [11]. Benefits observed with telephone appointments include two‐thirds of participants agreeing they save time, which agrees with smaller published studies prepandemic [18, 22]. An additional benefit may be a reduction in patients who ‘do not attend’ their scheduled appointments when telephone consultations are offered as observed in urology [28, 29]. A comparatively low number of our study participants had experienced online video appointments (9%); thus we recognize this lack of experience could influence the likelihood of participants reporting them as a preference. Trott et al. reported telephone and online video appointments were regarded as of equal value to in‐person appointments (65% and 62% respectively) by adults with CD [10]. Further study is required, ideally with a randomized controlled trial design to compare outcomes from in‐person, telephone or online video dietetic appointments for adults with CD.

While there is an increased offering of telephone and online video appointments, we observed a large proportion of patients still preferred in‐person appointments. In our study there was a trend towards a significantly greater proportion of older participants to prefer in‐person appointments; similarly, a recent United Kingdom study reported significantly fewer older patients considered telephone and online video appointments equally as useful compared with younger patients [10]. Our analysis highlights patients with a lower household income or those with no degree qualification were significantly more likely to prefer in‐person appointments, compared with telephone or online video appointments. This finding warrants further study to determine the reasons behind this, as previous studies have reported a decreased likelihood of internet access and use for health information among people with limited English proficiency, lower education levels, older age and lower‐income households [30].

The British Society of Gastroenterology recommendations highlight ‘…there will be some patients who do not have access to a telephone or appropriate information technology and therefore it is essential that accessibility to remote consultations is monitored to ensure that equality and diversity legislation is met’ [31]. We reported that 34% of participants who indicated they had a physical, sensory or cognitive disability, which made video/telephone communication difficult, have had a telephone/online appointment for CD, perhaps indicating inappropriate service provision. Patient (self) selection is key, with telephone or online video appointments available for those comfortable with the use of technology. It is noteworthy to highlight in the move towards greater digital healthcare, preliminary data have reported the benefits of an online prerecorded webinar, led by healthcare professionals, for adults newly diagnosed with CD [14]. By offering the webinar service it enabled dietitians to focus on utilizing their finite time where it was most needed for patients with lower health or digital literacy, minority ethnic groups where a translator was required and those with more complex health conditions. It is important that we listen to the needs of our patients in the development of services to ensure inclusivity and equality of care.

The majority of participants valued annual review appointments (irrespective of format), this being a similar proportion to recent United Kingdom and United States studies [14, 32, 33], with a further 12% unsure having not been offered an annual review in recent years. Healthcare provision is variable with many adults with CD not being offering healthcare reviews for CD in any format [14]. Approximately a third of patients with CD have recurrent or persistent symptoms despite being on a GF diet, and the most common cause is continued gluten ingestion [34]. A dietitian review of GF dietary adherence demonstrated that significantly fewer patients were following the GF diet compared with patients' self‐reported adherence [13, 35]; this healthcare intervention helped to identify involuntary gluten ingestion, improve symptoms and avoid repeat duodenal biopsies.

Our study presents data from a relatively large population of adults with CD, from a nonclinical setting, and thus captures people who are not regularly accessing healthcare support. A key strength of the study was that it reached a broad spectrum of adults with CD including those with less confidence in online technology. Through the inclusion of the paper survey, the study has captured the views of a more representative cohort of adults living with CD, compared with online data collection alone. Limitations to our study include the large proportion of Coeliac UK members due to recruitment procedures for the paper survey which may introduce responder bias; we also have a predominance of female participants and White ethnicity thus limiting the generalizability of findings. Targeted recruitment to ethnic minorities and the survey being available in languages other than English would have improved the study.

To conclude, it is important healthcare is available in an accessible format to optimize patient self‐management of the GF diet. It is important the views of patients with CD with differing health or digital literacy, minority ethnic groups and those who are not adhering to the GF diet or attending annual reviews, are heard, to enable the healthcare service to reach those in particular need.

## Author Contributions

**Yvonne Jeanes:** conceptualization, methodology, formal analysis, data collection, writing – original draft preparation, supervision, funding acquisition. **Lidia Orlandi:** methodology, formal analysis, data collection, writing – original draft preparation, funding acquisition. **Humayun Muhammad:** conceptualization, methodology, writing – review and editing, funding acquisition. **Sue Reeves:** conceptualization, methodology, writing – review and editing.

## Ethics Statement

The study was conducted in accordance with the Declaration of Helsinki and approved by the Institutional Ethics Committee of the University of Roehampton (LSC21/342).

## Conflicts of Interest

The authors declare no conflicts of interest.

### 1 Peer Review

The peer review history for this article is available at https://www.webofscience.com/api/gateway/wos/peer-review/10.1111/jhn.70014.

## Supporting information

Supporting information.

## Data Availability

Data available on request.
