# Supplementary material for: Telemedicine in Coeliac Disease: In‐Person Appointments Are Favoured by Patients With a Lower Education Attainment and Lower Household Income
Source: J Hum Nutr Diet. 2025 Jan 27;38(1):e70014. doi: 10.1111/jhn.70014 (PMC11773122; doi:10.1111/jhn.70014)
Supplement: Supplementary file 1 — Supporting information. [file JHN-38-0-s001.pdf]

YOUR VIEW: Telephone and online video appointments for coeliac disease

Introduction

Over the past year, healthcare appointments for coeliac disease have substantially moved away from face-to-face to telephone or online video consultations, due to the need to minimise the transmission of coronavirus. There are discussions underway as to what provision will be available in the future, partly informed by the success of provision during 2020/21.

It is important healthcare professionals hear the voice of people with coeliac disease regarding your experience and preference for appointments.

If you are not presently offered appointments for your coeliac disease, or you choose not to attend, your views on this issue are very important too.

The study findings will be presented to GPs, hospital doctors, and dietitians to inform the provision of appointments for people with coeliac disease.

THE STUDY AIMS to explore the acceptability of telephone and online video consultations in a UK population of adults with coeliac disease.

Your involvement:

The online questionnaire takes about 15 minutes. You can stop taking part in the survey

at any time. Please note that information provided before exiting the survey will be entered into the database.

Confidentiality:

All personal details will be kept confidential. A code number will be used to identify any information provided. Individual data will only be seen by the research team. No names will be used in any reports or publications related to this work.

Why take part in the survey?

To be part of research that aims to improve the healthcare provision for people with coeliac disease.

Additionally, all participants will be invited to take part in a prize draw, there are two £50 vouchers and two gluten free hampers to be won.

Lead Researcher:

Dr Yvonne Jeanes, Registered Dietitian

Health Science Research Centre, University of Roehampton, London SW15 4JD y.jeanes@roehampton.ac.uk Tel. 020 8392 3519

Research team:

Dr Humayun Muhammad, consultant gastroenterologist, Honorary Senior Research Fellow, University of Roehampton

Ms Lidia Orlandi, Masters of Research student, University of Roehampton

Consent statements

I agree to take part in this research, I am residing in the UK and have been diagnosed with coeliac disease, I am 18 years or older, and I am aware that I am free to withdraw at any point without giving a reason by contacting Yvonne Jeanes. I understand that if I do withdraw, my data may not be erased but will only be used in an anonymised form as part of an aggregated dataset. I understand that the personal data collected from me during the course of the project will be used for the purposes outlined above in the public

interest. By clicking on 'Yes, I consent' you are confirming that you have been informed about and understand the University's Data Privacy Notice for Research Participants. The information you have provided will be treated in confidence by the researchers and your identity will be protected in the publication of any findings. The purpose of the research may change over time, and your data may be re-used for research projects by the University in the future. ✱ *Required*

- ◯ Yes, I consent

The study has been approved by the procedures of the University of Roehampton Ethics Committee. If you have a concern about any aspect of your participation or any other query, please raise this with Yvonne Jeanes (y.jeanes@roehampton.ac.uk). However, if you would like to contact an independent party, please contact the Head of Department (Dr Caroline Ross, Department of Life Sciences, University of Roehampton, London SW15 4JD; Tel. no. 020 8392 3529; email c.ross@roehampton.ac.uk).

Should the Head of Department/ School change over the lifecycle of the research project the new Head of Department will become the independent contact. Contact details for the new Head of Department can be obtained from the investigator.

Full contact details will be available again at the end of the survey.

The survey is part funded by a Coeliac UK-sponsored dissertation award for Lidia Orlandi.

SECTION 1: BACKGROUND INFORMATION

This information is required to demonstrate the survey has reached a representative sample of adults living with coeliac disease.

What is your country of residence? (please tick the relevant option) ✱ *Required*

- ☐ England
- ☐ Scotland
- ☐ Wales
- ☐ Northern Ireland
- ☐ Other

If you selected Other, please specify:

Please indicate your sex: ✱ *Required*

Choose one option that best describes your ethnic group or background:

- ○ White
- ○ Asian/Asian British
- ○ Black/African/Caribbean/Black British
- ○ Mixed/Multiple ethnic groups
- ○ Other

If you selected Other, please specify:

Please indicate your current age in years:

Please indicate your highest level of education

- No formal qualifications
- GCSE/O level, apprenticeship or vocation level 2
- 'A' level or equivalent, vocational level 3
- University Degree(s)
- Other

If you selected Other, please specify:

What is your annual household income?

- less than £25,000
- £25,000 - £60,000
- above £60,000

- prefer not to say

Please indicate your time commitment during a usual working week: (you can tick more than one)

- ☐ Full-time paid work or full-time unpaid career
- ☐ Part-time paid work or part-time unpaid career
- ☐ Full-time education
- ☐ Long term sick or disabled
- ☐ Retired
- ☐ Looking after the family/home
- ☐ Unemployed
- ☐ Other

If you selected Other, please specify:

If you would like to add clarity to any of your answers, please do so here.

SECTION 2: COELIAC DISEASE

This section will ask you a few questions about coeliac disease and following the gluten-free diet.

Some of the questions may appear to be a little odd or appear to repeat themselves, these questions are from validated questionnaires.

Was your diagnosis of coeliac disease by a healthcare professional? ✱ *Required*

- Yes
- No
- Other

Approximately, what year were you diagnosed with coeliac disease? (eg 2005)

How would you rate your KNOWLEDGE of the gluten-free diet: ✱ *Required*

- Excellent
- Good
- Fair
- Limited
- Poor
- No knowledge

How CONFIDENT are you in managing your coeliac disease with a gluten-free diet? ✱ *Required*

- Very confident
- Fairly confident
- Neither confident nor unconfident
- Not confident
- Not at all confident

IN SHOPS/ SUPERMARKETS how confident are you in understanding food labels for a gluten-free diet? ✱ *Required*

- Very confident
- Fairly confident
- Neither confident nor unconfident
- Not confident
- Not at all confident

FROM ONLINE stores/ supermarket websites, how confident are you in understanding food labels for a gluten-free diet? ✱ *Required*

- Very confident
- Fairly confident
- Neither confident nor unconfident
- Not confident
- Not at all confident

Do you agree with the following statement "*The cost of gluten-free foods makes*

following a gluten-free diet harder for me"? ✱ *Required*

- Strongly agree
- Somewhat agree
- Neither agree nor disagree
- Somewhat disagree
- Strongly disagree

Do you agree with the following statement "*The COVID-19 pandemic substantially affected the management of my coeliac disease*"? ✱ *Required*

- Strongly agree
- Somewhat agree
- Neither agree nor disagree
- Somewhat disagree
- Strongly disagree

If you agree with the statement we would welcome your comments on how you have been affected:

Do you receive gluten-free foods on prescription?

- Yes
- No

If no, what is the reason for not receiving GF foods on prescription?

- Not available in my area
- Personal choice
- Other

Are you a member of Coeliac UK? ✱ *Required*

Quality of Life

How often have you experienced the following?

Have you been bothered by low energy levels during the past 4 weeks? ✱ *Required*

- None of the time
- A little of the time
- Some of the time
- Most of the time
- All of the time

Have you been bothered by headaches during the past 4 weeks? ✱ *Required*

- None of the time
- A little of the time
- Some of the time

- Most of the time
- All of the time

How strongly do you agree or disagree with the following statements?

I am able to follow a gluten-free diet when dining outside my home ✱ *Required*

- Strongly agree
- Somewhat agree
- Neither agree nor disagree
- Somewhat disagree
- Strongly disagree

Before I do something, I carefully consider the consequences ✱ *Required*

- Strongly agree
- Somewhat agree
- Neither agree nor disagree
- Somewhat disagree
- Strongly disagree

I do not consider myself a failure ✱ *Required*

- Strongly agree
- Somewhat agree
- Neither agree nor disagree
- Somewhat disagree

- Strongly disagree

Accidental gluten exposures

How important to your health are accidental gluten exposures? ✱ *Required*

- Very important
- Somewhat important
- Neutral/Unsure
- A little important
- Not at all important

Frequency of gluten exposure

Over the past 4 weeks, how many times have you eaten foods containing gluten on purpose? ✱ *Required*

- 0-never
- 1-2
- 3-5
- 6-10
- more than10

Do you eat gluten voluntarily? ✱ *Required*

- Yes
- No

If yes:

- A normal portion
- Just a taste

If just a taste:

- Often
- Rarely

When you eat out, do you tell the person who is cooking about your coeliac disease? ✱ *Required*

- Yes
- No

Do you check the labels of packaged foods? ✱ *Required*

- Yes
- No

Do you only eat packaged foods that are labeled gluten-free? ✱ *Required*

- Yes
- No

Do you consider annual review appointments for coeliac disease, in any format, important for you? ✱ *Required*

- Yes
- No
- Other

If you selected Other, please specify:

If you would like to add clarity to any of your answers, please do so here.

SECTION 3: TECHNOLOGY

The next few questions relate to what access you have to technology and the internet.

Do you have access to the internet at home, for example via a desktop, laptop or tablet? ✱ *Required*

- ○ Yes, I have good quality broadband
- ○ Yes, but broadband is inadequate for video calls
- ○ Yes, only with my smart phone contract
- ○ No

If you would like to add clarity to any of your answers, please do so here.

If yes: What electronic devices do you have access to at home? You can tick more than one

- ☐ Smartphone
- ☐ Desktop or laptop computer
- ☐ Tablet
- ☐ Other

If you selected Other, please specify:

How confident are you in using online technology? ✱ *Required*

- Very confident
- Fairly confident
- Neither confident or unconfident
- Not confident
- Not at all confident

Do you have any physical, sensory, or cognitive disabilities that make video/telephone conferencing difficult? ✱ *Required*

- Yes
- No
- Other, please provide details

If you selected Other, please specify:

SECTION 4: EXPERIENCE OF HEALTHCARE APPOINTMENTS– in person, telephone and online video appointments

Some of the questions may appear to repeat themselves, the questions are slightly different as we aim to capture the full range of healthcare appointments you have experienced.

When was your most recent appointment with a healthcare professional for managing the gluten-free diet/ coeliac disease ✱ *Required*

- Less than a year ago
- 1-2 years ago
- 2-5 years ago
- More than 5 years ago

Who was it with:

Was it:

- In person
- Via telephone
- Via online video consultation
- Other

If you selected Other, please specify:

Are you offered a review appointment, in any format, to help you manage the gluten-free diet/ management of coeliac disease? ✱ *Required*

- ◯ Yes, every year
- ◯ Yes, at least every 3 years
- ◯ Yes, but chose not to attend
- ◯ No
- ◯ Other (eg diagnosed less than a year ago)

If yes, who with:

Was it:

- ◯ In person
- ◯ Via telephone
- ◯ Via online video consultation
- ◯ Other

If you selected Other, please specify:

If you would like to add clarity to any of your answers, please do so here.

Please state the approximate distance to your last in-person appointment for coeliac disease (you can answer more than one).

- [ ] Hospital doctor
- [ ] GP
- [ ] Dietitian
- [ ] Pharmacist
- [ ] Other

If you selected Other, please specify:

Distance:

Have you attended a telephone or online video appointment for coeliac disease, or other health conditions, within the last 2 years? ✱ *Required*

- [ ] Yes, telephone appointment for coeliac disease
- [ ] Yes, online video appointment for coeliac disease
- [ ] Not for coeliac disease, I have had telephone appointment for a different condition
- [ ] Not for coeliac disease, I have had online video appointment for a different condition
- [ ] No telephone, nor online video appointment for any healthcare reasons
- [ ] Other

If you selected Other, please specify:

What was the reason?

- ○ Not offered a telephone nor online video appointment
- ○ Chose not to attend
- ○ Other

If you selected Other, please specify:

If you chose not to attend, which factors may have influenced your decision?

- ☐ I didn’t have the opportunity to schedule it
- ☐ I think it is not medically necessary
- ☐ I have poor access to technology
- ☐ I am not comfortable with technology
- ☐ I have privacy concerns
- ☐ I prefer in-person visits
- ☐ Other

If you selected Other, please specify:

If you have not been offered a telephone or online video appointment, would you like one to help you manage the gluten-free diet/ your coeliac disease in the future? (can tick more than one)

- [ ] Yes, online video appointment
- [ ] Yes, telephone appointment
- [ ] No
- [ ] Maybe

Any additional comments you would like to add about appointments for coeliac disease:

Please click on the statement most appropriate to your situation, this will enable you to be directed to the most relevant questions.

- ○ I have experienced a telephone or online video healthcare appointment
- ○ I have not had a telephone nor online healthcare appointment

Experience of telephone or online video healthcare appointment

If you have experienced a telephone or online video healthcare appointment please indicate your level of agreement with the statements below.

	1. Strongly disagree	2. Disagree	3. Neither agree nor disagree	4. Agree	5. Strongly agree
I was satisfied with the quality of the VIDEO during the online video appointment.	☐	☐	☐	☐	☐
I was satisfied with the quality of the SOUND during the online video appointment.	☐	☐	☐	☐	☐
I experienced technical difficulties during the online video appointment (e.g., unexpected disconnections, loss of sound or picture, etc.).	☐	☐	☐	☐	☐
The online video appointment made me feel nervous and uncomfortable.	☐	☐	☐	☐	☐
There were distractions within my home/ work environment during the online video appointment	☐	☐	☐	☐	☐

I could easily explain my medical problems to the healthcare professionals during the online video appointment	☐	☐	☐	☐	☐
I was confident that the healthcare profession could assess my condition via online video as if I was there.	☐	☐	☐	☐	☐
Online video appointment enables me to save money.	☐	☐	☐	☐	☐
Online video appointment enables me to save time.	☐	☐	☐	☐	☐
I have privacy concerns with online video appointments	☐	☐	☐	☐	☐
I was satisfied with the quality of the sound during the TELEPHONE appointment.	☐	☐	☐	☐	☐
The telephone appointment made me feel nervous and uncomfortable.	☐	☐	☐	☐	☐

There were distractions within my home/ work environment during the telephone appointment	☐	☐	☐	☐	☐
I could easily explain my medical problems to the healthcare professionals during the telephone appointment	☐	☐	☐	☐	☐
I was confident that the healthcare professional could assess my condition via telephone appointment as if I was there.	☐	☐	☐	☐	☐
Telephone call enables me to save money.	☐	☐	☐	☐	☐
Telephone call enables me to save time.	☐	☐	☐	☐	☐
I have privacy concerns with telephone appointments	☐	☐	☐	☐	☐

SECTION 5: Healthcare appointments for coeliac disease – your vision of the future

In this section, we would like to hear YOUR VIEW on what you would like to be offered for healthcare appointments.

What TYPE of appointment would you prefer to help you follow a gluten-free diet/ manage your coeliac disease in the future? eg. in person, telephone, online video...

What FREQUENCY of appointment would you prefer to help you to follow a gluten-free diet/coeliac disease in the future? eg once a year, once every 3 years, as and when I request etc...

WHO would you like to see for a coeliac disease review appointment?

- ☐ A hospital doctor/gastroenterologist
- ☐ A dietitian
- ☐ A dietitian with expertise in coeliac disease
- ☐ A general practitioner (GP)
- ☐ Pharmacist
- ☐ Other

Any additional comments:

Prize draw

Would you like to be entered into the prize draw, there are two £50 shopping vouchers and two gluten-free hampers to be won?

- Yes
- No

If you have answered yes, please supply your email address here:

We will only contact you in relation to the statement above that you have agreed to. Your email address will be removed once the prize draw has taken place.

If you would like to leave any comments relating to the survey, please do so here:

Final page

Thank you for taking the time to complete the survey.

Your information is valuable and will be an important contribution to informing the provision of future healthcare appointments for people with coeliac disease within the NHS.

Our contact details:

Lead Researcher: Dr Yvonne Jeanes, Registered Dietitian

Health Sciences Research Centre

University of Roehampton

London SW15 4JD

y.jeanes@roehampton.ac.uk

02083923519

The study has been approved by the procedures of the University of Roehampton Ethics Committee. If you have a concern about any aspect of your participation or any other query, please raise this with Yvonne Jeanes. However if you would like to contact an independent party, please contact the Head of Department (Dr Caroline Ross, Department of Life Sciences, University of Roehampton, London SW15 4JD; Tel. no. 020 8392 3529 ; email c.ross@roehampton.ac.uk).

Key for selection options

3 - Please indicate your sex:

- Male
- Female
- Prefer not to say
- Other

19 - Are you a member of Coeliac UK?

Yes

No

No, though I have been previously
